# Noninvasive reconstruction of internal heat source in biological tissue using adaptive simulated annealing algorithm

**DOI:** 10.1038/s41598-024-67253-w

**Published:** 2024-07-16

**Authors:** Fuli Ye, Diwen Shi, Cheng Xu, Kaiyang Li, Minyue Lin, Guilian Shi

**Affiliations:** 1 School of Biomedical Engineering and Imaging, Xianning Medical College, Hubei University of Science and Technology, Xianning, 437100 China; 2 School of Physics and Technology, Wuhan University, Wuhan, 430072 China

**Keywords:** Health care, Energy science and technology, Engineering, Mathematics and computing

## Abstract

The heat distribution information of human lesions is of great value for disease analysis, diagnosis, and treatment. It is a typical inverse problem of heat conduction that deriving the distribution of internal heat sources from the temperature distribution on the body surface. This paper transforms such an inverse problem of bio-heat transfer into a direct one, thereby avoiding complex boundary conditions and regularization processes. To noninvasively reconstruct the internal heat source and its corresponding 3D temperature field in biological tissue, the adaptive simulated annealing (ASA) algorithm is used in the simulation module, where the position *P(x, y, z)* of point heat source in biological tissue and its corresponding temperature *T* are set as the optimization variables. Under a certain optimized sample, one can obtain the simulated temperature distributing on the surface of the module, then subtract the simulated temperature from the measured temperature of the same surface which was measured using a thermal infrared imager. If the sum of absolute values of the difference is smaller, it indicates that the current sample is closer to the true location and temperature of the heat source. When the values of optimization variables are determined, the corresponding 3D temperature field is also confirmed. The simulation results show the simulated position and temperature of the heat source are very approximate with those of the real experimental module. The method presented in this paper has enormous potential and promising prospects in clinical research and application, such as tumor hyperthermia, disease thermal diagnosis technology, etc.

With the rapid development of engineering thermophysics, researches on human biological heat transfer phenomena has gradually shifted from qualitative to quantitative^1,2^. The noninvasive reconstruction of 3D temperature field in biological tissue is very valuable for the further development of biomedical engineering field^3,4^. It is a typical heat conduction inverse problem that how the heat sources and the interior temperature field distribution are deduced through the surface temperature of living body^5^. The equation set formed for describing this kind of heat conduction inverse problems are often ill-posed, which doesn’t mean they are unsolvable, but the solving process is very cumbersome^6,7^. The solution of inverse problem often goes against the natural order of physical processes, so fundamentally, many good properties in direct problem are no longer satisfied in inverse one. The inverse problem of heat conduction often faces the difficulty of nonlinearity, especially when introducing multi-parameter models analysis, which requires a huge amount of computation^8^. The traditional analytic methods can be hardly used in solving or analyzing the ill-posed problems. Even if the analytic methods are stiffly adopted, the solutions perhaps have no any realistic significance because of the great distance between the theoretical values and the real ones^7–9^. So the ill-posed problems often need a processing of regularization to make the obtained solutions more approximate to the real status^10^. Nowadays, the traditional numerical methods such as boundary element method (BEM), finite element method (FEM), and finite difference method (FDM) are commonly used in the modeling and simulation of bio-heat conduction inverse problems^11–13^. However, these methods are very strict with geometric profile of the simulation object, and need heavy computation^14^. Radial basis neural networks have advantages such as strong nonlinear fitting ability, simple learning rules, compact topology structure, and fast convergence speed. However, the interpretability of this method is poor, and it cannot work when the data is insufficient, making it difficult to determine the number of hidden layer nodes, node centers, and widths, which can lead to pathological data in the optimization process^15,16^. Many mesh free methods do not have a sound mathematical theoretical foundation, and are still in the exploratory stage in terms of convergence, stability, and error estimation, which makes it difficult to solve problems in engineering applications. In fact, mesh free methods do not have advantages in computational efficiency. The shape function in finite element method uses a unified polynomial function, which is convenient and simple. However, most mesh free methods require shape functions for points, which may be different and complex according to different points, resulting in a huge amount of computation for the corresponding matrix^17–19^. For these reasons, the numerical methods mentioned above are not suitable for the the modeling and simulation of bio-heat conduction reverse problems on account of the complexity of biological tissue and organs, and also face great difficulties in the clinical applications such as the real-time detection of temperature field in lesions and its’ surrounding tissues during tumor hyperthermia, the thermal analysis and diagnosis of diseases^20^. In our preliminary work, we used the traditional numerical methods such as FEM, BEM and FDM to simulate the 3D temperature field of biomimetic materials respectively, and conducted comparative analysis regarding the simulation results^21,22^. In our further researches, we used parallel particle swarm optimization (PPSO) algorithm and multi-island genetic algorithm (MIGA) to simulate 3D temperature field in high-purity polypropylene material whose thermal properties are similar with the adipose tissue. The results showed that both the PPSO and the MIGA are suitable for the simulation in resolving the reconstruction of internal heat sources in biomimetic materials, and the simulation efficiency and accuracy were significantly improved compared to other optimization methods^23^. In the heat conduction analysis model of this paper, the information of the internal heat source and its corresponding temperature field were derived through the surface temperature of the experimental model, so the boundary condition belongs to the first type one, namely Dirichlet boundary condition^13^.

Based on optimization platform of software ISIGHT, a novel method of acquiring the distribution information of heat source and the 3D temperature field adopting ASA algorithm is presented in this paper. Different heat sources are corresponding to different temperature field distribution, and there exists an only mapping between them. Based on the basic heat conduction theory mentioned above, the position *P(x,y,z)* and its corresponding temperature *T* of point heat source in biological tissue are supposed as the optimization variables in this research. Due to the excellent global search performance of ASA algorithm, the process of searching for the optimal values are accelerated efficiently. After determining the position and temperature of the heat source, its corresponding 3D temperature field is also determined accordingly. On account of the method and the optimization model presented in this paper, a heat-conduction reverse problem is transformed to be a direct one, which greatly reduces the complexity in solving such inverse heat conduction problems. The work in this paper possesses a significant and practical meaning in the clinical field such as tumor hyperthermia, disease thermal diagnosis technology, etc.

## Methods

### Basic theory and operational steps of ASA algorithm

Simulated annealing algorithm (SAA), widely used in various application fields on account of its advantages such as high optimization quality, strong robust, easy to realize and so on, is a random searching algorithm which is developed from the procedure of hot working of mental^24^. In the process of resolving practical optimization problems, it is found that the computing time of SAA often needs a long time, and the optimization efficiency is also relatively low^25^. In order to shorten the searching time and improve the optimization efficiency, the fast simulated annealing algorithm is developed. However, this improved algorithm based on SAA just processes the controlling parameter *T* by promoting the its descent speed in a simple way, and such a modification does not have reasonable theory foundation^26^. The SAA generally takes the Metropolis function as the acceptance criterion to avoid falling into the trap of local optimum, which need a long time to achieve the heat balance in procedure of annealing under a certain temperature. The adaptive simulated annealing (ASA) algorithm can adaptively adjust parameters and strategies based on the current searching state, thereby improving the global optimization ability and computational efficiency of the algorithm. So the ASA is especially suitable for the the research of complex inverse problems such as the reconstruction of the internal heat source in biological tissue. Compared with the traditional SAA, the improvements of ASA algorithm mainly involves the following aspects^27–30^:

#### Generating function of new variables

Disturbing the variables on account of the theory of self-adaptive neighborhood. The disturbance mode is shown as Eq. (1).

1 $$ j = i + \gamma m_{u} $$

Here *i* is the optimization variable, *m*_*u*_ is the self-adaptive step length, *j* is the new variable after disturbing and *r* is the Cauchy distribution of stochastic disturbance. The region of search can be express as follow:

2 $$ G_{c} (x) = \frac{T(t)}{{T^{2} (t) + x^{2} }} $$

In Eq. (2), *G*_*c*_(*x*) is the region of search, x denotes some point in the neighborhood interval. It can be known from this equation that when the temperature is at a low point the algorithm will search in the adjacent domains of current model, and otherwise when the temperature is at a high point the algorithm will perform a large-scale search in solution space. There is a smooth trailing in the Cauchy distribution, and such a characteristic makes the algorithm possess the ability of quick jumping out of the local optimum. The generating function of new variables is much faster in convergence rate after adding the Cauchy distribution.

#### Probability of acceptance after adopting Metropolis criterion

The probability of acceptance is shown as Eq. (3):

3 $$ P = \exp \left( { - \frac{f(j) - f(i)}{{T_{k} }}} \right) $$

where *f(i)* and *f(j)* are the objective function values of optimization variable and new variable after disturbing respectively. If *f(j)* < *f(i)*, the new solution will be accepted with probability 1, and this new solution is also the optimal solution of problem. Conversely, the acceptance probability of new solution given according to Eq. (3). When the algorithm begins working, the value P is relatively large, so the probability of accepting deteriorative solution is also high. At the end stage of the performing procedure of algorithm, the current solution approaches to the global optimum, the value P will decrease gradually and converge at the global optimum with full probability.

#### Cooling mode

The cooling mode is shown as Eq. (4).

4 $$ T_{k} = T_{0} \exp ( - CK^{\frac{1}{N}} ) $$

Here, C is a given constant coefficient, T_0_ is the initial temperature, K is the iterative number of temperature and N is number of parameters. Generally, Eq. (4) can be transformed to Eq. (5) which is shown as follow:

5 $$ T_{k} = T_{0} \alpha^{{K^{\frac{1}{N}} }} $$

where the value range of α is 0.7 ≤ α ≤ 1.

### Establishment of objective function

In the optimization model, the position *P(x, y, z)* of internal point heat source and its corresponding temperature *T* are set as the optimal variables. All possible positions in the model and their temperature values form the whole sample space. According to the basic theory of heat conduction, each sample corresponds to a certain surface temperature distribution, which is the basis for constructing the objective function. Under a certain optimized sample, one can obtain the simulated temperature distributing on the surface of the module, then subtract the simulated temperature from the measured temperature of the same surface which was measured using a thermal infrared imager. If the absolute value of the difference is smaller, it indicates that the current sample is closer to the true location and temperature of the heat source. When the values of optimization variables are determined, the corresponding 3D temperature field is also confirmed. The objective function is expressed as follow:

6 $$ f_{\min } (x,y,z,t) = \sum {\left| {T_{n} - T_{En} } \right|} $$

In Eq. (6), *T*_*n*_ and *T*_*En*_ are the simulated temperature and the experimental temperature respectively of a same point on surface, *n* denotes the number of points. By the iterative calculation method shown as Eq. (6), one can determined the optimal position and its corresponding temperature value of point heat source, and then the distribution information of 3D temperature field in biological tissue can be acquired accordingly. It is typical heat conduction reverse problem how the internal temperature field distribution and the heat source distribution are derived from the surface temperature. This paper presents a optimal model that can exactly transform the reverse problem to a direction one, avoiding the tedious processing procedure of traditional numerical method such as regularization and boundary condition processing. In most practical cases, the surface temperature is often taken as the boundary condition (the first boundary condition), which means one has to firstly obtain all the surface temperature data in traditional numerical methods. The processing is complex or even unacquirable as far as the real living organism is concerned. The optimization method and model presents in this paper overcomes the difficulties above successfully, and just needs limited surface temperature data which can be easily obtained with thermal infrared imager.

In this research, a novel method of establishing objective function is proposed in the optimization model to reduce the computation of searching for the optimal solution during the optimization process. In the infrared thermal image of experimental module, two straight lines that intersect vertically are set, and then some equidistant points are set on the lines (the number of points can be reasonable set according to the actual size of module). One can extract the temperature values of these points, and subtract them with the simulated temperature values of the corresponding points under a certain sample (a position of point heat source and its corresponding temperature in space of samples), then acquire the sum of absolute value. This method is not only simple and practicable, but also avoiding the complex establishing process of objective function without affecting the precision of algorithm, and the optimization speed is promoted greatly. In regard to the analysis of heat source distribution and temperature field distribution, such a method has a high theoretical and practical value. So, Eq. (6) can be modified as follow:

7 $$ f_{\min } (x,y,z,t) = \sum {\left| {T_{1n} - T_{E1n} } \right|} + \sum {\left| {T_{2n} - T_{E2n} } \right|} $$

where *T*_*1n*_ and *T*_*2n*_ respectively denote the simulated temperature values of equidistant points chosen on the two orthogonal straight lines which are set on a certain surface of simulation model, *T*_*E1n*_ and *T*_*E2n*_ respectively denote the experimental temperature values of the chosen equidistant points on two orthogonal straight lines that are set on the experiment model. The lines and the points on the experiment model are perfectly mapping to those on the simulated model.

### Reconstruction of 3D temperature field

Equation (7) is taken as the objective function to perform the iterative computations. When the objective value *f*_*min*_ reaches to the minimum , the current sample (including the position of heat source and the corresponding temperature value) is just the optimal results. After determining the position and temperature of the heat source, its corresponding 3D temperature field in biological tissue is also determined accordingly. By such a method, a bio-heat conduction reverse problem is transformed to be a direct one. The finite element analysis software ANSYS and the typical Pennes bio-heat conduction model are adopted in our simulation. ANSYS is a large finite element analysis software which contains three typical heat conduction modes, and can perform thermal simulation and analysis under various complex circumstance. It is commonly recognized that Pennes bio-heat conduction equation is the best model for the analysis of bio-heat, which can be expressed as follow ^31^:

8 $$ \rho {\text{c}}\frac{{\partial {\text{T}}}}{{\partial {\text{t}}}} = \nabla ({\text{k}} \cdot \nabla {\text{T}}) + {\text{w}}_{{\text{b}}} \rho_{{\text{b}}} {\text{c}}_{{\text{b}}} ({\text{T}}_{{\text{a}}} - {\text{T}}) + {\text{Q}}_{{\text{m}}} $$

In Eq. (8), *T* (*x, y, z*) is the distribution function of internal temperature field, ρand c denote the density (Kg/m^3^) and the thermal capacitivity (J /Kg·℃) of biological tissue respectively, w_b_, ρ_b_ and c_b_ denote the blood perfusion rate (ml/s·ml^-1^), blood density (Kg/m^3^) and blood thermal capacitivity (J /Kg·℃) respectively, and Q_m_ is the rate of metabolic heat production per unit volume of tissue (W/m^3^). In this research, the vitro pork adipose tissue was used as the experimental model in which the metabolic process was almost stagnant, therefore the item Q_m_ was omitted in our analysis.

### Experiment and data processing

#### Experimental material

The pork adipose tissue, whose density is 827 kg/m^3^ and thermal conductivity is 0.1265 W/(m·K), is adopted in the experiment of bio-heat conduction^32^. The size of pork adipose tissue is 7.5 cm × 2.4 cm × 3.0 cm, two resistors (15.5Ω and 30.5Ω) are adopted as the internally installed heat sources, and the diameter of 4 wires is 0.5mm. The temperature measuring tool of model surface is non-refrigerated focal plane infrared thermal imager whose temperature resolution is 0.05℃, pix is 640 × 480, and focusing range is 0.5m ~ ∞. The high precision digital point-thermometer JM222, whose probe can reach to the very position of pork adipose tissue model where the resistors are placed, is adopted to detect the temperature of heat source.

#### Experimental process

**Figure 1:**
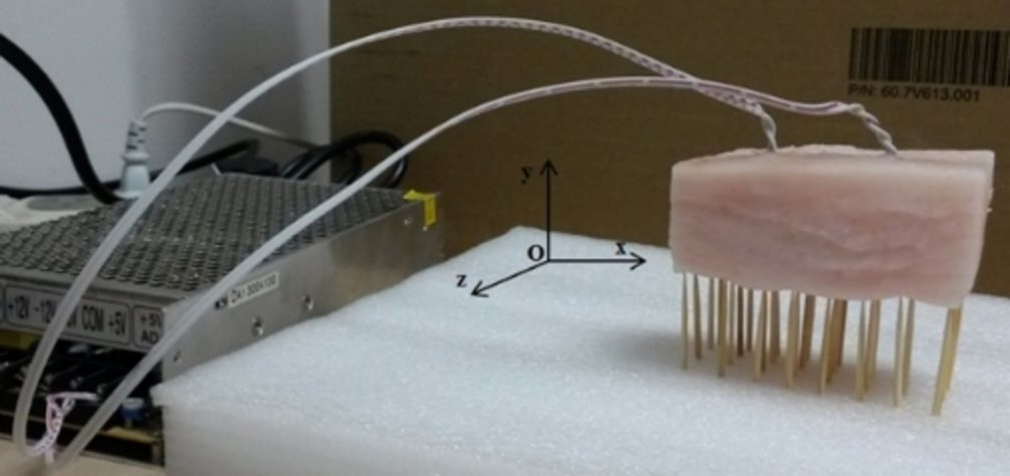
Connection of pork adipose tissue and its support.

The pork adipose tissue was incised to a cuboid form, taking the bottom rear corner on the right side of the pork adipose module as the origin to establish rectangular coordinate. So, according to the measurement of the pork adipose tissue, the length of the cuboid in *x* direction was 7.5 cm, the height in *y* direction was 2.4 cm, and the width in *z* direction was 3.0 cm (Shown as Fig. 1). Using a slim knife, two incisions were made on the upper surface(*y* = 2.4 cm) of the cuboid. The specific coordinates of the deepest point of one incision were *x* = 2.5 cm, *y* = 1.7 cm, *z* = 1.6 cm, meaning the depth of this incision was 0.7 cm, and those of the other incision were *x* = 5.6 cm, *y* = 1.5 cm, *z* = 1.4 cm, meaning the depth was 0.9 cm. A resistor(30.5Ω) was inserted into the adipose tissue module from the incision whose depth was 0.7 cm, reaching to deepest point; Another resistor(15.5 Ω) was inserted into the adipose tissue module from the incision whose depth was 0.9 cm, reaching to the deepest point. Two resistors were connected with a 5V power supply. In order to ensure the six faces of adipose tissue module to be free for heat conduction and keep the shape as non-deforming as possible, some wooden toothpicks were adopted and aligned to be an array for supporting the adipose tissue module. When the two resistors were powered on, they began to generate heat and transferred it to the surface of the adipose tissue module. The surface temperature was detected using a infrared imager which was connected with a PC machine. When the heat conduction reached equilibrium, an infrared thermal image of the surface of the adipose tissue module at z = 3.0 cm was taken, in which the temperature data were extracted as fitness comparison data for optimizing iterative calculations. Meanwhile, the probe of digital point-thermometer was plugged to the position where the resistor located through the incision in the adipose tissue module, and the actual temperature value of heat source could be acquired. Based on the principle of fluid mechanics, when the airflow velocity V ≤ 0.15 m/s and the environment temperature is 18 ~ 20℃, the heat conduction of object surface in air belongs to laminar natural convection. Therefore, in the experiment procedure, the environment temperature was controlled at 19.4℃, the windows and doors was kept close. In such circumstances, the laminar natural convection was set as 3.368 W/m^2^·K^33^.

#### Experimental data and processing

**Figure 2:**
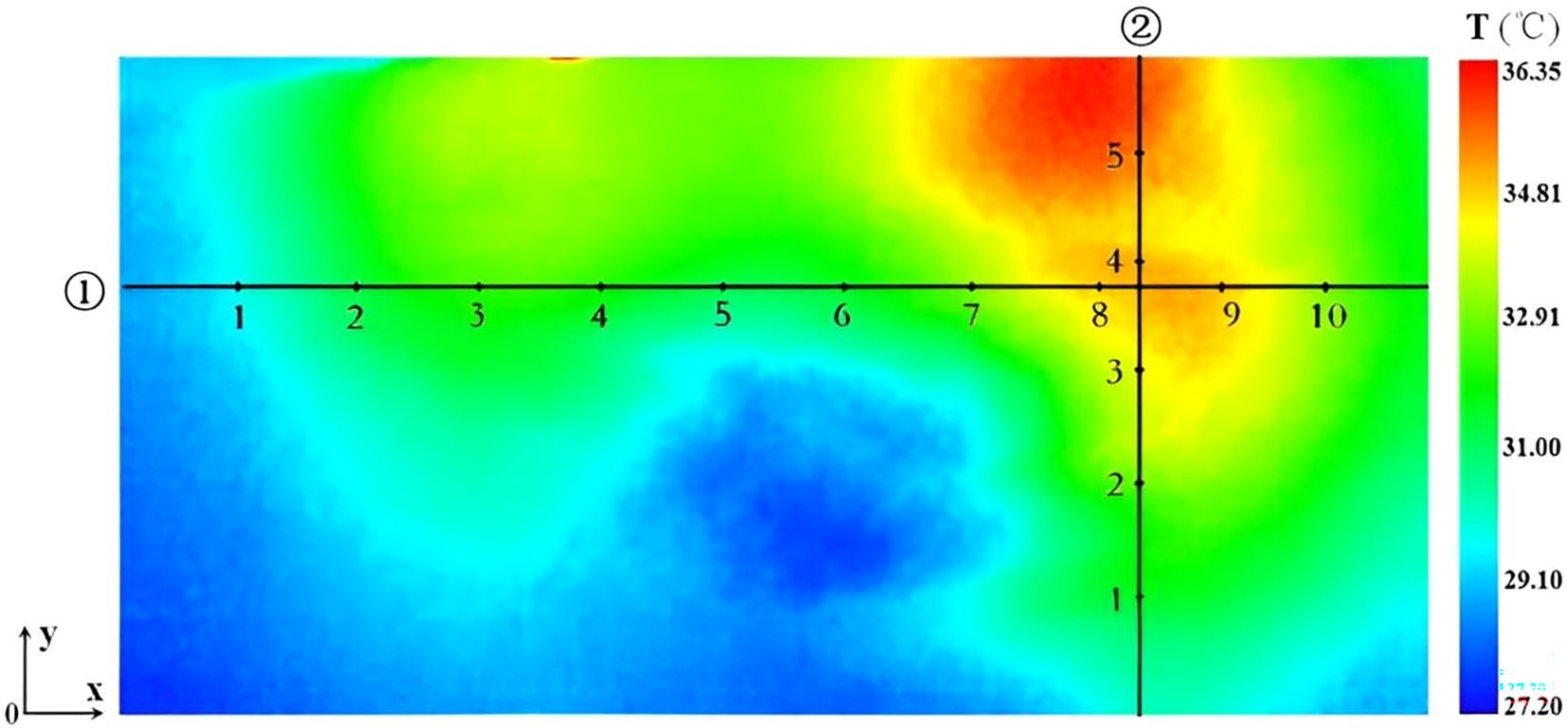
Two straight lines and equidistant points acquired from the surface z = 3.0 cm of pork adipose tissue module.

A specialized temperature data processing software of infrared detector was adopted to extract the temperature values of 15 points which were distributed on two orthogonal straight lines on the surface z = 3.0 cm of adipose tissue module (Shown as Fig. 2). The specific position of two straight lines were ① *y* = 1.7 cm, *z* = 3.0 cm and ② *x* = 5.0 cm, *z* = 3.0 cm, respectively. Ten equidistant points were set on line ① and five equidistant points were set on line ②. The coordinate values of these 15 points and their corresponding temperature were given in Table1. From theoretical aspect, the two orthogonal straight lines could be chosen arbitrarily. However, in actual operation, the two orthogonal straight lines should be set through the high-temperature region of thermal image as far as possible, which meant that temperature distribution characteristic of surface could be reflected via the 15 equidistant points. The temperature values of two resistors at their middle position, measured with a point-thermometer, were 60.75℃ and 84.40℃ respectively. In fact, when the temperature of two resistors reached to a relatively high value, the adipose tissue around the resistors melted in a very small scale, which exerted some influence on the surface temperature distribution of experimental model. However, compared with the whole adipose tissue module, the proportion of melted adipose tissue was very small, which meant errors caused by the melted adipose tissue could be ignored. In practical procedure, the melted adipose tissue was regarded as a part of the heat source.

**Table 1:** Coordinate values of 15 equidistant points from surface *z* = 3.0 cm of the pork adipose tissue module and their corresponding temperature values.

Equidistant points	Straight Line ①			Straight Line ②		
	*x*/cm	*y*/cm	T/℃	*x*/cm	*y*/cm	T/℃
1	0.68	1.70	29.82	5.00	0.40	29.24
2	1.36	1.70	31.98	5.00	0.80	30.28
3	2.05	1.70	33.06	5.00	1.20	31.95
4	2.73	1.70	33.22	5.00	1.60	34.02
5	3.41	1.70	32.75	5.00	2.00	35.65
6	4.09	1.70	31.79	–	–	–
7	4.77	1.70	33.55	–	–	–
8	5.45	1.70	34.75	–	–	–
9	6.14	1.70	34.21	–	–	–
10	6.82	1.70	33.02	–	–	–

## Simulation procedure and results

### Establishment of ANSYS parameterized model

The heat conduction model of pork adipose tissue module was established with ANSYS parametric design language (APDL), which is the precondition that the ISIGHT automatically calls ANSYS and implements optimization procedure for acquiring the optimal heat source position and corresponding temperature distribution. APDL is a kind of scripting language affiliated to ANSYS itself. As a powerful tool and method, this language makes the application software ANSYS more convenient and perfect, and can automatically implement the finite element analysis^23,24^. Using APDL, the setting of parameters and the building of simulation model can be accomplished automatically, and also different analysis type can be chosen freely according to the need. Here, the size of simulation model was set as *x* = 7.5 cm, *y* = 2.4 cm, *z* = 3.0 cm, the density was set as 827 kg/m^3^ and the thermal conductivity was 0.1265 W/(m·K). The environment temperature was set as 19.4 ℃, and the surface convective heat transfer coefficient was 3.368 W/m^2^·K.

### Simulation procedure and results

**Figure 3:**
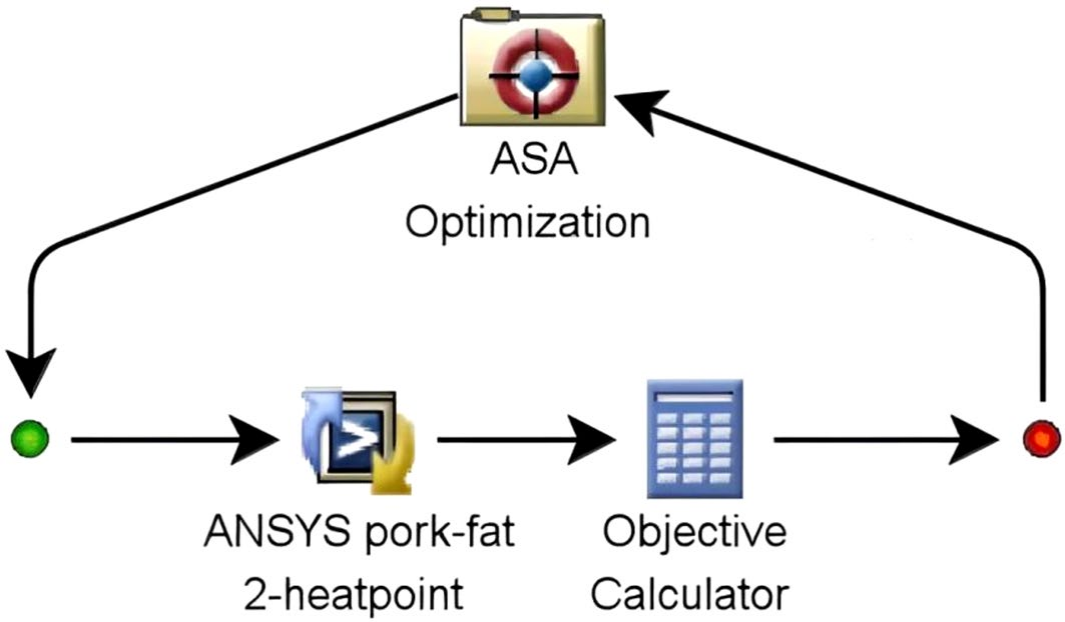
ISIGHT flow diagram of reconstruction of heat sources in pork adipose tissue based on ASA algorithm.

ISIGHT is a multidisciplinary optimization design platform, and commonly used in the engineering design field at present. It is developed out by Engineous Software Company of America based on Unix platform and Windows NT^34^. This software organically integrates design exploration technologies, reasoning technologies and digital technologies, and the functions are greatly expanded after the comprehensive exploitation. ISIGHT can also integrate multi-domain simulation code and afford corresponding intelligent support, realizing the evaluation and study to different design alternatives^35^. Such a process can greatly shorten the period of product design and promote the work efficiency. The ISIGHT simulation procedure of reconstruction about the 3D temperature field was shown as Fig. 3.

In Fig. 3, the left dot denoted the beginning of computational process and the right one denoted the end computational process. The data transmission among the three data processing module was unidirectional. First, The module “ASA Optimization” output the initialized variables to the module “ANSYS pork-fat 2-heatpoint”. Then the module “ANSYS pork-fat 2-heatpoint” substituted the variables into the FEM model and worked out the result under a certain individual sample. The result was transmitted to the module “Objective Calculator”in which the calculation program of objective function was stored, and the module “Objective Calculator” could automatically work out the objective function value of each individual sample. Finally, as the evaluation basis of corresponding individual sample, this value was transmitted to the optimization module “ASA Optimization” and the optimization module determined whether the replacement of a new sample was necessary according to the optimal settings such as the number of iterations, the setting of minimum value of objective function. In order to reduce the calculated amount and promote the optimization efficiency, a suitable objective function was established in the module “Objective Calculator”. The calculation of objective value need combine the temperature data of 15 equidistant points on two straight lines which were set on the thermal image. The objective function in the “Objective Calculator” module was shown as Eq. (9).

9 $$ \begin{gathered} f_{\min } = \left| {T_{11} - 29.82} \right| + \left| {T_{12} - 31.98} \right| + \left| {T_{13} - 33.06} \right| + \hfill \\ \begin{array}{*{20}c} {\begin{array}{*{20}c} {} & {} \\ \end{array} } & \begin{gathered} \left| {T_{14} - 33.22} \right| + \left| {T_{15} - 32.75} \right| + \left| {T_{16} - 31.79} \right| + \hfill \\ \left| {T_{17} - 33.55} \right| + \left| {T_{18} - 34.75} \right| + \left| {T_{19} - 34.21} \right| + \hfill \\ \left| {T_{110} - 33.02} \right| + \left| {T_{21} - 29.24} \right| + \left| {T_{22} - 30.28} \right| + \hfill \\ \left| {T_{23} - 31.95} \right| + \left| {T_{24} - 34.02} \right| + \left| {T_{25} - 35.65} \right| \hfill \\ \end{gathered} \\ \end{array} \hfill \\ \end{gathered} $$

Here, T_1n_ (1 ≤ n ≤ 10) and T_2m_ (1 ≤ m ≤ 5), the temperature values of 15 equidistant points on two straight lines, were from the module “Objective Calculator”. The calculation result f_min_ was transmitted to the module “ASA Optimization”. The batch file was compiled and called into the module “ANSYS pork-fat 2-heatpoint”. The number of iterations was set as 5500 times.

**Figure 4:**
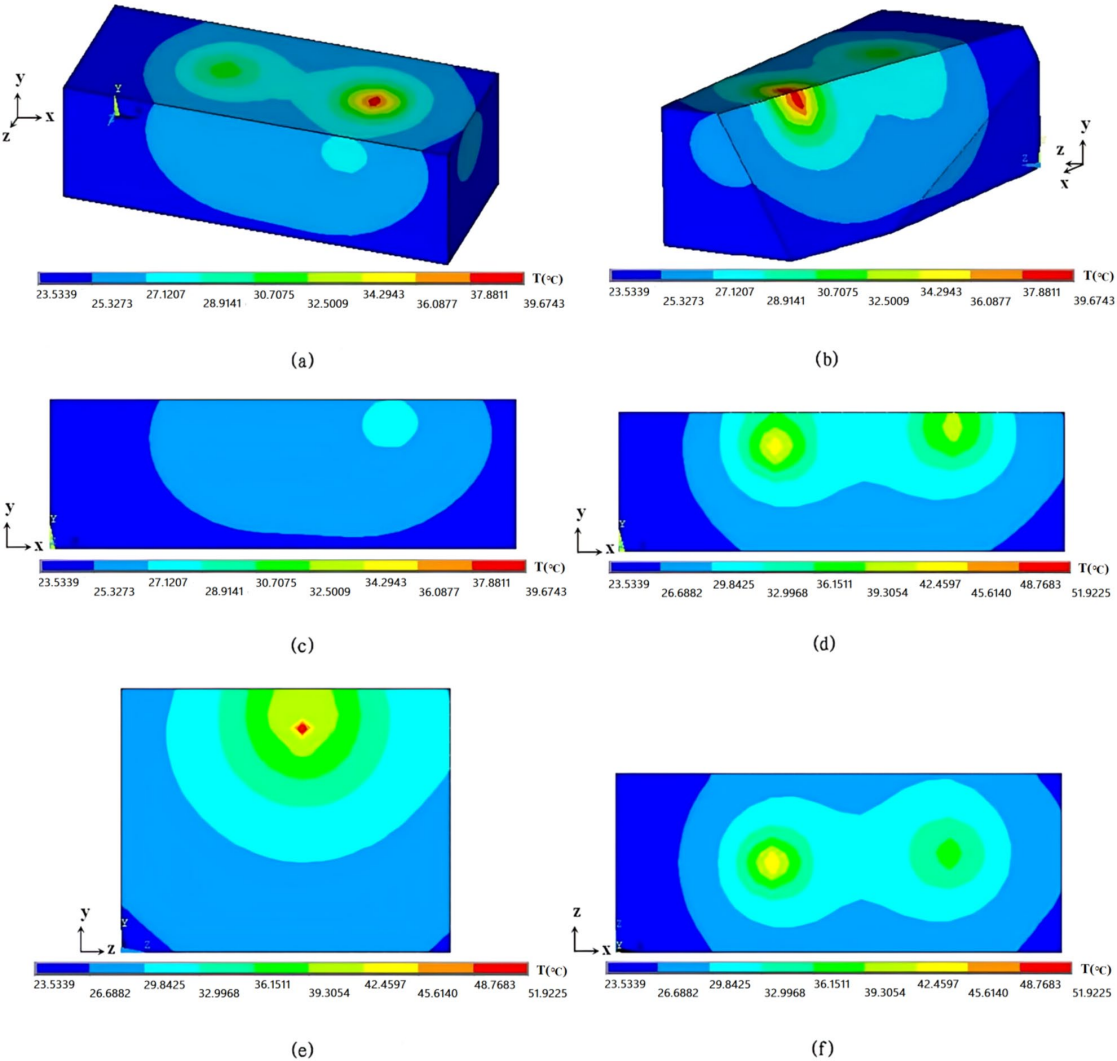
Simulation results of 3D temperature field of pork adipose tissue based on ASA algorithm. (**a**) The full view of surface temperature distribution. (**b**) The temperature distribution of arbitrary section of adipose tissue module. (**c**) The temperature distribution of surface z = 3.0 cm. (**d**) The temperature distribution of section z = 1.3 cm. (**e**) The temperature distribution of section x = 5.2 cm. (**f**) The temperature distribution of section y = 1.5 cm.

After 5500 iterations, the optimal simulation position corresponding to the 30.5 Ω resistor heat source was (2.52 cm, 1.68 cm, 1.63 cm) and the one corresponding to the 15.5 Ω resistor heat source was (5.54 cm, 1.53 cm,1.43 cm). The simulation temperature values corresponding to the two heat sources ware 86.33 ℃and 62.15 ℃, respectively. The detailed simulation images were shown as Fig. 4. Here, Fig. 4a was the surface temperature distribution diagram of cuboid adipose tissue in a whole field of view. Figure 4b was the temperature distribution diagram of arbitrary section, which indicated one could acquire any interested temperature distribution section of adipose tissue module. Figure 4c showed the temperature distribution of surface z = 3.0 cm, which was very approximate to the infrared thermal image of the same surface. Figure 4d–f were the temperature distribution sections that were parallel with the surfaces from three directions, and the corresponding sections were *z* = 1.3 cm, *x* = 5.2 cm and *y* = 1.5 cm respectively. These three sectional views could well incarnate the distribution characteristics of internal heat from different angles, and also reflect the status of internal heat sources. The temperature in adipose tissue module was higher than that of surface, so in the temperature distribution sections, the area where the temperature was beyond the temperature scale of color strip appeared gray, which did not contain any temperature meaning. In order to solve this problem, the temperature scale of color strip was reset through adjusting the values of “Min contour value” and “Max contour value” in the software ANSYS. In Fig. 4a – c, a original color strip was used, and in Fig. 4d–f, the color strip was adjusted to a higher temperature scale.

## Discussion and analysis

Comparing the experimental temperature values with the corresponding simulation ones, it is found that the simulation results acquired with ASA algorithm are higher than those acquired with the infrared imager. The experimental and simulation temperature values of 15.5Ω resistor are 60.75℃and 62.15℃ respectively, with the error of 2.31%; The experimental and simulation temperature values of 30.5Ω resistor are 84.40℃ and 86.33℃respectively, with the error of 2.29%. As for the position of heat sources, the experimental and simulation position of 15.5Ω resistor are (5.6 cm, 1.5 cm, 1.4 cm) and (5.54 cm, 1.53 cm,1.43 cm) respectively; and those of 30.5Ω resistor are (2.5 cm, 1.7 cm, 1.6 cm) and (2.52cm, 1.68 cm, 1.63 cm) respectively. The comparing results show there is no significant difference between the positions measured in the experiment and those calculated in the simulation. The detailed comparison between the experimental and simulated values is shown in Table 2.

**Table 2:** Comparison between experimental and simulated values.

	Location of Resistor (30.5Ω)			Location of Resistor (15.5Ω)			Temperature of Resistor (30.5Ω)(℃)	Temperature of Resistor (15.5Ω)(℃)
	x ( cm)	y ( cm)	z ( cm)	x ( cm)	y ( cm)	z ( cm)		
Simulation values	2.52	1.68	1.63	5.54	1.53	1.43	86.33	62.15
Measured values	2.5	1.7	1.6	5.6	1.5	1.4	84.40	60.75
Error							2.29%	2.31%

From the perspective of experimental model, experiment procedure and simulation model, the reasons of temperature error and position difference are analyzed as following: (1) The real biological tissue (pork adipose) is adopted in the experimental model, the thermophysical parameters of biological tissue will change correspondingly with the increase of its temperature. In the experiment procedure, the adipose tissue around the two resistors is melted in a small range, which is a main cause of temperature error of heat sources. It is seen from the results that the higher experimental temperature of heat sources will lead to the relatively significant error (When the experimental temperature is 60.7℃, the error is 2.4%, and when the experimental temperature is 84.0℃, the error is 2.8%). (2) In order to insert the resistors into the adipose tissue module, a narrow knife is used to make two incisions in different depths. There's still some heat coming out of the gap although the knife’s width has been controlled to a reasonable scope. This is an another reason that causes errors. (3) The electrified wires can also create heat, which is equivalent of line heat sources set in the adipose tissue model. Compared with the heat created by the resistors, the heat created by the electrified wires is much smaller, but it is still an important factor that causes errors. (4) Because of the characteristics of biological soft tissue, the pork adipose model can be only controlled to be an approximate cuboid. But in the simulation process, the constructed model is set to be a standard 3D cubiod one. (5) The biological structures of pork adipose tissue is relatively complex, and the heat transfer in experiment model is anisotropic. In the simulation model, it is difficult to set the thermophysical parameters of adipose tissue to their true state, which is also an important reason leading to errors. From an algorithmic perspective, the core of ASA algorithm is the decrease of temperature. With the decreasing of temperature, the probability of accepting inferior solutions gradually decreases and eventually approaches zero. However, if the temperature decreases too slowly, the algorithm will need more time to reach the lower temperature, which means the algorithm needs more iterations and the time cost will also increase. Another core of the ASA algorithm is its ability to accept worse solutions, which is the only way for the algorithm to escape from local optima. However, when the iteration falls into a local optimal solution, the probability of the algorithm being awakened to other regions is small. Even if the algorithm gets stuck in a poor local optimal solution, it still takes a long time to jump out. Therefore, it is necessary to find a suitable balance between reduction speed of temperature and the probability of accepting inferior solutions, and increase the diversity and randomness in jumping out of local optimal solutions. From the perspective of selection operation of surface temperature data, the 15 points are chosen equidistantly on two vertically intersecting straight lines, and temperature distribution characteristic of surface should be reflected via the temperature values of the 15 equidistant points as far as possible. In fact, there is still a certain degree of randomness in the setting of two vertically intersecting straight lines and the selection of equidistant points on the surface. So, different selection operation of surface temperature data can lead to different comparing values in the target function of ASA algorithm, which may cause different influence to the simulation results.

## Conclusions

In this research, the real biological tissue is adopted in the experimental model. To investigate the noninvasive reconstruction of the internal heat source and the corresponding 3D temperature field, the ASA algorithm and the large-scale finite element analysis software ANSYS are introduced in the simulation process, in which a suitable objective function is designed to fasten the iterative calculation. In the experiment part, two different resistors are used as the internal heat sources, and the related data acquired with the infrared imager are extracted for the computation and comparison in the objective function of optimization algorithm. In the simulation part, the APDL program is compiled to automatically perform the modeling, meshing, heat source setting, finite element calculation and temperature data acquiring of related nodes. It is shown from the results that the simulation distribution of surface temperature is consistent with the principle of heat transfer, and the simulation temperature values are slight above the experimental ones. In consideration of the environmental factors, measuring error factors and simulation parameter setting factors, the errors between the experiment results and simulation ones are within a reasonable range. This reconstruction method of 3D temperature field that combines the global optimization algorithm and the FEM is convenient and fast, and possesses broad application prospects in medical engineering field. The research in this present paper plays a positive role in promoting the development of therapeutic and diagnostic instruments, biology, medicine, environment, and new materials.

## Data Availability

All the data generated and analyzed during this study are included within this published article, and its supplementary information is available via this link (https://doi.org /10.6084/m9.figshare.25511752).
